# Seroprevalence of human brucellosis among the tribal and non-tribal population residing in an eastern state of India: Findings from the state-wide serosurvey

**DOI:** 10.3389/fmicb.2022.1070276

**Published:** 2022-11-28

**Authors:** Debaprasad Parai, Subrat Kumar Sahoo, Matrujyoti Pattnaik, Aparajita Swain, Annalisha Peter, Lopamudra Jena Samanta, Rashmita Pradhan, Hari Ram Choudhary, Kanhu Charan Nahak, Sanghamitra Pati, Debdutta Bhattacharya

**Affiliations:** Department of Microbiology, ICMR-Regional Medical Research Centre, Bhubaneswar, India

**Keywords:** Odisha, ELISA, IgG antibody, seroprevalence, brucellosis

## Abstract

**Background:**

Brucellosis is a neglected zoonotic disease found predominantly in lower- and middle-income countries (LMICs), causing significant public health concern in India. The objective of this study was to assess the prevalence of human brucellosis in Odisha, India among community members involved in animal husbandry as a common practice.

**Method:**

This cross-sectional study included 817 adult participants from 11 districts in Odisha. Four districts from the Northern division, four districts from the Central division, and three districts from the Southern division were selected for the study. Blood samples were collected during a COVID-19 serosurvey in Odisha conducted from 1st to 17th September 2021. Immunoglobulin-G (IgG) antibodies were measured against *Brucella* using a commercial ELISA kit. Point estimates at 95% confidence intervals (CIs) and adjusted odds ratio were calculated.

**Results:**

The overall prevalence of anti-Brucella IgG antibodies was calculated at 16.65% (95% CI: 14.19–19.42). The highest seropositivity was found in Sambalpur district (29.73%; 95% CI: 16.43–47.16) and the lowest was determined in Mayurbhanj district (4.44%; 95% CI: 0.99–15.60). Compared to males, females were more prone to contracting the disease (AOR: 1.13; 95% CI: 1.05–1.67). Participants from rural settings had higher prevalence of anti-Brucella IgG antibodies than urban dwellers (AOR: 4.53; 95% CI: 1.73–11.86).

**Conclusion:**

This study revealed that human brucellosis was associated with sociodemographic factors like gender, living settings, and household numbers. To prevent brucellosis, screening should be initiated, infected humans should be treated early, and the public should be educated about risk factors and preventive measures.

## Introduction

Brucellosis is a major neglected zoonotic disease and is among the most widespread zoonoses found predominantly in low- and middle-income countries (LMICs), where it is responsible for substantial health, economic, and livelihood burdens (Hull and Schumaker, 2018; World Health Organization, 2020). *Brucella melitensis* is the most virulent species that causes infections in humans, whereas bovine and caprine brucellosis are primarily caused by *Brucella abortus* and *Brucella melitensis* (Corbel, 2006; Hull and Schumaker, 2018). The disease has an adverse effect on both human and animal health and a significant socioeconomic impact on the rural population, which mostly depends on livestock rearing and livestock-related activities for daily wages (Etemadi et al., 2020; Lindahl et al., 2020). Brucellosis affects a broad variety of wild and domestic animals, causing infertility, repeat breeding, retention of placenta, abortions, and even lower milk production, which results in enormous economic losses for livestock (McDermott et al., 2013). Contact with animal fluids, intake of raw dairy products, and ingestion of undercooked meat are the main causes of transmission in humans (Kang et al., 2014). *Brucella* can cause both acute and chronic illnesses in humans, but it remains misdiagnosed due to its non-descript clinical presentation in humans (Mantur and Amarnath, 2008; Kang et al., 2014). Human brucellosis has a non-specific and highly variable clinical presentation. Symptoms such as undulant fever, chills, headache, arthralgia, and myalgia are frequently present in patients with brucellosis. The disease is also linked to splenic abscess, spondylitis, endocarditis, renal failure, orchitis, and encephalitis (Buzgan et al., 2010; Dagli et al., 2011; Zhong et al., 2013). Farmers, veterinarians, slaughterhouse workers, and livestock keepers are at a greater risk of infection because of their professional interactions with livestock (Pappas et al., 2006; Dean et al., 2012).

Approximately 500,000 global cases of brucellosis occur in humans annually, although the exact number should be higher because of under-reporting (Hull and Schumaker, 2018). Despite these numbers, it remains a significant disease burden in LMICs, as the disease does not receive proper attention from health systems. As a result, the World Health Organization (WHO) currently lists brucellosis as one of the top neglected zoonoses (WHO, 2011). In the Mediterranean region of Europe, Africa, Central, South, and Middle East Asia, and Central and South America, it is a significant disease for humans (Abou, 2000; Igawe et al., 2020).

An estimated 80% of Indians live in close proximity to domestic or wild animals, putting them at risk for brucellosis (Mantur and Amarnath, 2008). On the other hand, domestic animals and dairy products are irreplaceably linked to the livelihood of Indian rural communities. Therefore, people who act as animal handlers are always at a greater risk of contracting brucellosis because of their constant chances of exposure to an infected animal (Proch et al., 2018). Few studies have measured the prevalence of human brucellosis, mostly among veterinary professionals in different states of India such as Karnataka, Punjab, Maharashtra, and Assam, ranging from 2.4 to 55.0% (Yohannes and Gill, 2011; Mangalgi et al., 2015; Shome et al., 2017; Jamir et al., 2020; Mangtani et al., 2020; Ghugey et al., 2021). A single study performed in Odisha in 2013 to address human brucellosis seroprevalence among high-risk groups determined a maximum seroprevalence of 9.09% (Priyadarshini et al., 2013). To understand the impact of human brucellosis and to develop sustainable control strategies, it is essential to determine its distribution and frequency.

The purpose of this research was to determine the prevalence of anti-Brucella IgG antibodies in 11 districts of Odisha. This epidemiological study aimed to estimate the disease burden of human brucellosis in this state to develop more effective management and control measures.

## Methodology

### Study setting

The Eastern state of Odisha lies between latitudes of 17°49’ N and 22°34’ N and longitudes of 81°27′ E and 87°29′ E. Its geography is characterized by river basins, plateau regions, hills, and coastal plains. As a tropical savannah area, it has a hot, humid climate (annual average of 70–75%), high temperatures (average of 26–43°C in summer), medium rain (1,400–1,600 mm annually), and mild winters (average of 13–28°C). With 22.85% of the state’s population being tribal, it has the third-highest percentage of tribals in the country. It is estimated that nearly 83% of Odisha’s population lives in rural areas, and most of their income comes from agriculture and livestock farming. Forests cover approximately 37.34% of Odisha’s land, making it one of the most forested states in the country (State profile, 2022).

### Study design

Sera were separated from 4 ml of blood, collected from adult (age ≥ 18 years) participants who took part in the Odisha COVID-19 serosurvey which was conducted from 1st to 17th September 2021. A population-based cross-sectional design was adopted for this survey based on the sampling framework used in the national serosurvey by the Indian Council of Medical Research (ICMR; Murhekar et al., 2020). A multi-stage random sampling method was used, where clusters within districts were selected proportionate to size, and households within clusters were selected systematically by random sampling. A subset of the samples was chosen based on their job profile, socioeconomic status, and sociodemographic status, and further tested for anti-Brucella IgG antibodies. Samples were transported to the laboratory, maintained in a cold chain, and stored at −20°C for further serological analysis of brucellosis. Demographic details of the participants were collected using electronic devices with an open data kit (ODK) tool.

**Figure 1:**
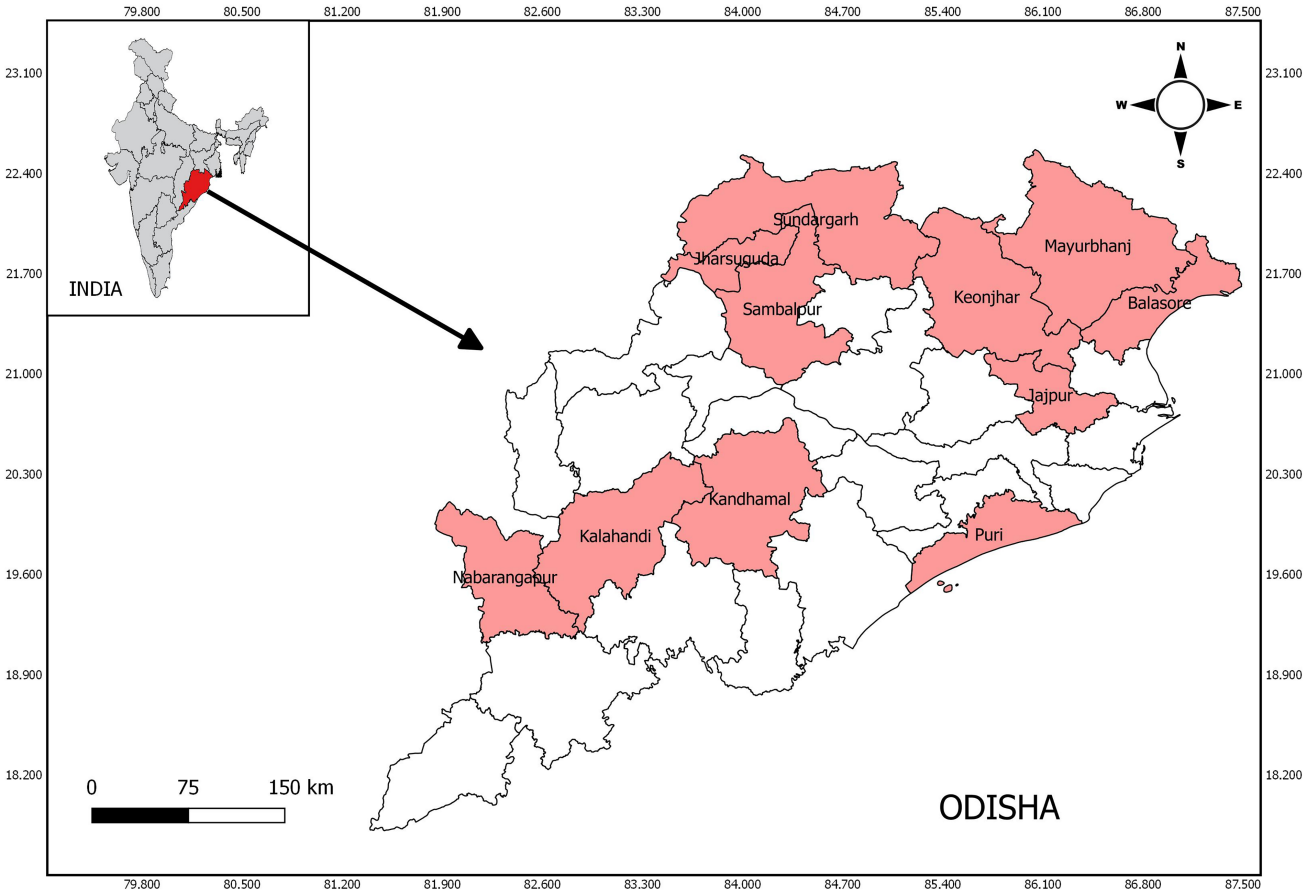
Study sites of human brucellosis in Odisha, India.

A total of 817 samples were selected from 11 districts in three revenue divisions (Northern, Central and Southern) of the state (Figure 1). Four districts were selected from the Northern division: Jharsuguda (53; 6.49%), Sundargarh (85; 10.40%), Keonjhar (71; 8.69%), and Sambalpur (37; 4.53%); four districts were from the Central zone: Mayurbhanj (45; 5.51%), Balasore (28; 3.43%), Puri (22; 2.69%), and Jajpur (14; 1.71%); three districts were from the Southern zone: Nabarangpur (210; 25.70%), Kandhamal (139; 17.01%), and Kalahandi (113; 13.83%).

### Ethical approval

All participants were asked to provide written informed consent before participating in the study. The study was conducted with the approval of the State Ethical Committee of Odisha and the Institutional Ethical Committee of ICMR – RMRC, Bhubaneswar.

### Serological investigation

An enzyme-linked immunosorbent assay (ELISA) was performed to determine the presence of anti-Brucella IgG antibodies using a commercial kit (Brucella IgG ELISA; Cat No. BA052G) following the manufacturer’s protocol (Calbiotech Inc., CA, USA). In brief, 200 μl of sample diluent was added to 10 μl of sera samples at a ratio of 1:21, and then 100 μl diluted samples were dispensed into a 96-well ELISA plate and incubated at room temperature for 20 min. Washing was performed three times with 300 μl of 1X wash buffer, 100 μl enzyme conjugate was dispensed into each well and further incubated for 20 min at room temperature. The enzyme conjugate was removed after incubation and the wells were washed. The 3,3′,5,5′-Tetramethylbenzidine (TMB) substrate solution (100 μl) was added to the plate and incubated for another 10 min at room temperature. Finally, 100 μl of stop solution was added to the reaction, and the optical density (OD) was read at 450 nm using an automated microplate ELISA reader. The antibody index was calculated using a given formula. The interpretation was as follows: <0.9 = no detectable antibody; 0.9–1.1 = borderline positive; >1.1 = detectable IgG antibody to *Brucella*.

### Statistical analysis

Statistical analyses were performed using SPSS software version 21.0 (SPSS Inc., Chicago, IL). Descriptive analysis and 95% confidence intervals were calculated for the variables. Univariate and multivariate analysis was performed using logistic regression to calculate the unadjusted odds ratio (UOR) and adjusted odds ratio (AOR). The value of *p* ≤ 0.05 was considered statistically significant. Choropleth map was generated using QGIS (v3.18).

## Results

Among 817 participants from 11 districts, most (*n* = 462; 56.55%) were from the Southern division, followed by the Northern (*n* = 246; 30.11%) and the Central (*n* = 109; 13.34%) divisions. The Southern division contributed most participants, as the majority there involved either in livestock handling or in agriculture. The sociodemographic characteristics of the participants are presented in Table 1. Most (*n* = 445; 54.46%) of the participants were aged between 18 and 45 years and the majority (*n* = 502; 61.44%) were male. Almost 80% of the participants came from tribal groups in Odisha.

**Table 1:** Sociodemographic characteristics of study participants.

Variables	Frequency	Proportion (%)
*Age*		
18–45	445	54.47
45–60	231	28.27
Above 60	141	17.26
*Gender*		
Male	502	61.44
Female	315	38.56
*Caste*		
General	60	7.34
Other Backward Castes	71	8.69
Schedule Caste	32	3.92
Schedule Tribe	654	80.05
*Marital status*		
Unmarried	88	10.77
Married	700	85.68
Widowed	29	3.55
*Household members*		
01-Mar	155	18.97
04-Jun	518	63.4
More than 6	144	17.63

Sera samples of 54 (6.61%) participants was determined as borderline positive for IgG antibodies against human brucellosis. Sundargarh district had the highest percentage (10.58%) of borderline positive cases and the lowest was measured in Balasore district (Table 2). A total of 136 (16.65%) participants had anti-Brucellosis IgG antibodies index above 1.1 and considered as true positive for human brucellosis (Table 2). Hereafter, seropositive means only true positive samples. The highest prevalence of anti-Brucella IgG antibodies (29.73%) was found in the Sambalpur district, while the lowest of 4.4% was calculated for the Mayurbhanj district. Division-wise, the highest IgG antibodies to *Brucella* was found in the participants from the Northern districts (20.73%) compared to 16.45 and 8.25% in the Southern and Central districts, respectively.

**Table 2:** Prevalence of anti-Brucella IgG antibodies in the study districts.

District	Number of sample (*N*)	Borderline IgG positive (%)	95% CI	IgG positive (%)	95% CI
Balasore	28	0	–	2 (7.14)	1.24–24.95
Jajpur	14	1 (7.14)	0.68–34.12	1 (7.14)	0.68–34.12
Jharsuguda	53	3 (5.66)	1.61–16.25	5 (9.43)	3.52–21.42
Kalahandi	113	9 (7.96)	3.93–14.98	19 (16.81)	10.67–21.26
Kandhamal	139	14 (10.07)	5.82–16.62	31 (22.30)	15.86–30.30
Keonjhar	71	2 (2.81)	0.48–10.71	12 (16.90)	9.40–28.05
Mayurbhanj	45	2 (4.44)	0.99–15.60	2 (4.44)	0.99–15.60
Nabrangpur	210	11 (5.24)	2.77–9.42	26 (12.38)	8.39–17.79
Puri	22	1 (4.54)	0.67–22.43	4 (18.18)	5.99–41.00
Sambalpur	37	2 (5.40)	1.32–18.25	11 (29.73)	16.43–47.16
Sundargarh	85	9 (10.58)	5.25–19.61	23 (27.06)	18.25–37.96
Total	817	54 (6.61)	5.04–8.59	136 (16.65)	14.19–19.42

Prevalence of anti-Brucella IgG antibodies was 17.08, 14.72, and 18.44% in the 18–45 years, 45–60 years and >60 years age categories, respectively. Brucellosis seropositivity was highest among the age group above 60 years, while the lowest anti-Brucella IgG was found in the 45–60 years age group. However, the adjusted odds ratio (AOR) was calculated as 0.99 (CI: 0.57–1.74) for >60 years compared to participants from 18 to 45 years of age.

Almost 19.37% of females had anti-Brucella IgG antibodies and it was 1.13 times more than males, as calculated by AOR (Table 3). Seropositivity was higher in the schedule caste (21.88%) and schedule tribe (17.28%) groups than in other ethnic groups (12.21%). The infection history was prevalent among participants from a household size above six persons, and the calculated AOR showed that they had 1.56 times more probability of having anti-Brucella IgG antibodies in comparison to participants from a household size below three members. Seropositivity was 14.19, 16.99, and 18.06% among members from 1 to 3 households, 4–6 households and more than 6 households, respectively. The percentage of IgG against *Brucella* was only 5.77% in urban residences compared to 18.23% in rural areas. Participants from rural settings were more likely to have human brucellosis than their urban counterparts (AOR: 4.53; 95% CI: 1.73–11.86).

**Table 3:** Sociodemographic risk factors associated with human brucellosis.

Variables	Frequency	IgG positive	95% CI	UOR (95% CI)	AOR (95% CI)
*Age*					
18–45	445	76	13.76–20.97	Ref	Ref
45–60	231	34	10.54–20.10	0.84 (0.54–1.30)	0.78 (0.49–1.24)
Above 60	141	26	12.60–26.03	1.10 (0.67–1.79)	0.99 (0.57–1.74)
*Gender*					
Male	502	75	11.99–18.43	Ref	Ref
Female	315	61	15.23–24.25	1.36 (1.02–1.87)	1.13 (1.05–1.67)
*Ethnicity*					
General	60	8	6.33–25.14	Ref	Ref
OBC	71	8	5.33–21.53	0.82 (0.29–2.35)	0.58 (0.17–1.98)
SC	32	7	9.94–40.44	1.82 (059–5.58)	0.59 (0.14–1.49)
ST	654	113	14.50–20.44	1.35 (0.62–2.93)	0.45 (0.13–1.61)
*Household size*					
01-Mar	155	22	9.29–20.91	Ref	Ref
04-Jun	518	88	13.91–20.56	1.23 (0.74–2.05)	1.38 (0.98–2.35)
>6	144	26	12.33–25.52	1.33 (0.71–2.47)	1.56 (1.04–3.00)
*Residence*					
Urban	104	6	2.36–12.63	Ref	Ref
Rural	713	130	15.50–21.30	3.64 (1.56–8.48)	4.53 (1.73–11.86)

## Discussion

Brucellosis is a major concern worldwide, but remains a neglected disease that poses significant health, economic, and livelihood challenges in LMICs (Franc et al., 2018). It is most prevalent among livestock handlers because they frequently come in contact with diseased animals. Additionally, families of these groups face a high risk of possible domestic exposure due to the proximity of animals in residential spaces. The inadequacies of healthcare in LMICs like India are exacerbated by socioeconomic factors, with brucellosis most often affecting marginalized and poor communities (Dean et al., 2012).

A variety of factors have contributed to a continuous change in the epidemiology of human brucellosis over the last 25 years, including extensive livestock farming, sanitary conditions, and socioeconomic factors (Pappas et al., 2006; Shome et al., 2017; Delam et al., 2022). Several climatic variables such as rainfall, temperature, relative humidity, and sunshine duration influence seasonal fluctuations in the transmission of human brucellosis (Yang et al., 2020). Hence, to formulate targeted control measures against human brucellosis, it is necessary to analyze the epidemic situation, demographic features, seasonal data, incidence rate in that region, geographical distribution, and genetic profiles of *Brucella* isolated in this province (An et al., 2021).

In our study, we found a greater proportion of anti-Brucella IgG seropositive female participants than male adults, and the significance was pronounced, corroborating the findings of Nematollahi et al. (2017). Most studies have shown that seroprevalence of human brucellosis was higher among males than females due to more involvement in animal husbandry (Alkahtani et al., 2020; Ghugey et al., 2021; Mehari et al., 2021). However, those studies were mostly on veterinarians, para-veterinarians, and professional animal handlers, as compared to our study participants, predominantly from the tribal community members. Here, females are more susceptible to brucellosis because household responsibilities, such as rearing livestock, are usually assigned to females in tribal areas. There may also be limitations in access to healthcare facilities among females owing to gender-related differences.

We observed that participants from a big family of more than six members were associated with the occurrence of human brucellosis. This could be explained by poor socioeconomic status, lack of maintenance of personal hygiene and protection, proper sanitisation and a clean living environment (Kothalawala et al., 2017; O'Callaghan, 2020). Prevalence of higher anti-Brucella IgG antibodies among the participants aged >60 years are likely due to their traditional roles in livestock care and management. This finding is in agreement with global studies, in which elderly individuals were more likely to be infected with brucellosis (Rahman et al., 2012; Tumwine et al., 2015). Changes in job responsibilities from domestic animal husbandry to personal business or services in the modern era among younger people could lead to the lower seropositivity in their age categories. Residents in rural settings were at a higher risk of brucellosis than urban residents, as found in our study. Brucellosis seroprevalence was almost five times higher in rural communities than in urban counterparts. Similar data have been found in most Asian and African countries with incidences of human brucellosis (Tumwine et al., 2015; Golshani and Buozari, 2017; Munyua et al., 2021; Tao et al., 2021). This can be explained by poverty among rural people, close contact with domestic animals, lack of awareness and consumption of raw dairy products. A similar study in Iran identified that contact with livestock, animal husbandry, and farming were among the significant risk factors of human brucellosis (Keramat et al., 2020). Central division of Odisha had the lowest anti-Brucella IgG seropositivity compared to Southern and Northern districts. A possible reason for this variation could be due to the geographical distribution of backward ethnic groups (scheduled castes, scheduled tribes, and other backward castes), which are primarily concentrated in the Northern and Southern divisions. An overall 6.61% of borderline positive cases could be alarming, however, clinical follow-up and further testing are recommended for those cases before reaching to a conclusion.

Underreporting and insufficient monitoring of data, lack of financial resources, and cooperation between veterinarians and human medics often lead to misconceptions regarding the true incidence of human brucellosis. Data completeness and representativeness can only be improved when disease priority is defined by individual countries and funds are reallocated to national surveillance. A greater diagnostic capacity would lead to a reduction in misinterpretation and diagnostic delays, and would facilitate rapid and effective treatment. Veterinary practice should incorporate animal handlers into training programs for biosecurity measures conducted through government efforts. Increasing awareness and improving disease control practices should facilitate changes in the perception of brucellosis vaccination in animals.

This study had a few limitations. First, as the blood samples were collected during COVID-19 serosurvey, availability of in-depth demographic and symptomatic data is limited. Second, in addition to the seropositive samples, other clinical findings were not considered. Lastly, the lack of molecular or culture methods to confirm brucellosis cases could limit the overall prevalence.

## Conclusion

The study is the first of its kind to estimate human brucellosis seroprevalence among community members across a state in India. The findings will provide necessary inputs for planning and implementing intervention strategies in the region. The lack of data and underreporting of human brucellosis cases in LMICs makes it difficult to determine the precise seroprevalence of this disease. Increasing awareness of brucellosis among the general public and healthcare professionals through health education campaigns is essential. The current data also provide a basis for more robust surveillance programmes to establish the epidemiological characteristics of human brucellosis in Eastern India.

## Data availability statement

The original contributions presented in the study are included in the article/supplementary material, further inquiries can be directed to the corresponding authors.

## Ethics statement

The studies involving human participants were reviewed and approved by State Ethical Committee of Odisha and the Institutional Ethical Committee of ICMR – RMRC, Bhubaneswar. The patients/participants provided their written informed consent to participate in this study.

## Author contributions

SP and DB conceptualized, planned, and formulated the study. LS, HC, and KN were involved in data collection and interpretation. DP, SS, AS, AP, and RP were responsible for laboratory testing. MP and DP did the data analysis. DP, SS, and MP prepared the initial draft. SP and DB supervised the study. All the authors have read and approved the final version of the manuscript.

## Funding

The study was carried out using the intramural funds received from Indian Council of Medical Research, Govt. of India.

## Conflict of interest

The authors declare that the research was conducted in the absence of any commercial or financial relationships that could be construed as a potential conflict of interest.

## Publisher’s note

All claims expressed in this article are solely those of the authors and do not necessarily represent those of their affiliated organizations, or those of the publisher, the editors and the reviewers. Any product that may be evaluated in this article, or claim that may be made by its manufacturer, is not guaranteed or endorsed by the publisher.
